# Swine *Trichinella* Infection and Geographic Information System Tools

**DOI:** 10.3201/eid1407.071538

**Published:** 2008-07

**Authors:** Robin Burke, Penny Masuoka, K. Darwin Murrell

**Affiliations:** * Uniformed Services University of the Health Sciences, Bethesda, Maryland, USA

**Keywords:** trichinellosis, public health, *Trichinella spiralis*, GIS, pigs, organic, pasture, reservoir hosts, dispatch

## Abstract

Pastured pigs are vulnerable to *Trichinella spiralis* infection through exposure to wild reservoir hosts. To evaluate the potential impact of the expanding production of pork from pasture-raised pigs, we mapped locations of *T. spiralis* occurrence and pastured-pig farms in the United States. Twenty-eight farms were located within 50 km of previous infection.

The incidence of *Trichinella spiralis* infection in humans and swine has declined markedly in North America over the past 20 years; however, sporadic outbreaks still occur (*1*,*2*). The importance of sylvatic reservoir hosts in the persistence of *T. spiralis* infection risk is well-documented, even in countries that have made substantial gains in controlling the infection in swine (*2*–*5*); *T. spiralis* infection has been recently demonstrated in foxes in Ireland, where no pig infections had been identified for 30 years (*6*). The outdoor rearing of pigs is a major risk because of increased exposure to sylvatic and synanthropic hosts (*2*–*10*). Transmission of *T. spiralis* from infected farm pigs to synanthropic (e.g., rats, cats, raccoons) and local sylvatic animal populations also occurs (*3*,*11*).

Pastured-pig operations in the United States have experienced substantial growth in recent years. The number of pigs reared in organic livestock operations, which by law must pasture pigs for at least some part of the day, rose from 1,724 in 2000 to 10,018 in 2005 (*12*). An even larger number of pigs (>100,000) are now being reared nonorganically on pasture and marketed as “pastured, humane, or free-range” pigs. (See below for source of information.) Because of the sporadic occurrence and distribution of outbreaks, and the lack of routine monitoring, the impact of this increase on the risk for *T. spiralis* infection for pastured farm swine is unknown. We report the use of geographic information system (GIS) methods to locate potential high-risk foci to facilitate targeting of surveillance for domestic pig infections, similar to the recent study identifying areas of risk for fascioliasis (*13*).

## The Study

Two *Trichinella* databases (All Hosts and Domestic Pig) were compiled by using literature published over the past 60 years (full list of references provided on request from kdmurrell@comcast.net) and *Trichinella* isolate records from North America, maintained at the International *Trichinella* Reference Center in Rome (www.iss.it/site/Trichinella). The All Hosts database contains records on *T. spiralis* infections in wildlife, including synanthropic species such as rats, cats, skunks, and foxes. The second database, Domestic Pig, contains records on *T. spiralis* from domestic pigs. The sylvatic species *T. nativa* and *T. murrelli,* which occur in North America, are not infective for pigs (*Sus scrof*a). *T. pseudospiralis*, which has low infectivity for pigs, has been reported only from a vulture and from a wild boar in North America, but because of the wide range of the former species (12,000–18,000 ha) and the location of wild boars >150 km from a known pastured-pig operation, we excluded this species from our analysis. When latitude and longitude data on host collection sites were not available, we approximated the locations using the coordinate points of the closest town to the collection site. From the 201 *T. spiralis* records that were collected, 54 were selected for mapping (37 wildlife hosts and 17 domestic pig infections). Other records were eliminated either because of vague descriptions of location or because they could not be confirmed as *T. spiralis* rather than a sylvatic species. The infected sylvatic hosts included black bear (*Ursus americanus*), raccoon (*Procyon lotor*), opossum (*Didelphis virginiana*), feral pig/wild boar (*Sus scrofa*), red fox (*Vulpes vulpes*), gray fox (*Urocyon cinereoargenteus*), feral cat (*Felis catus*), striped skunk (*Mephitis mephitis*), coyote (*Canis latrans*), and mink (*Neovison vison*). With the exception of black bears, these wild animals are potentially synanthropic hosts and transfer *T. spiralis* between the sylvatic and domestic habitats (*2*–*5*).

A third database was created for US farms that raise organic or nonorganically pastured swine. We obtained these data by searching the Internet using the keywords “pasture,” “pork,” and “organic” for farms producing and marketing pork through the Internet. The latitude/longitude coordinates from town and state data were determined by using the website www.zipinfo.com/search/zipcode.htm.

**Figure 1:**
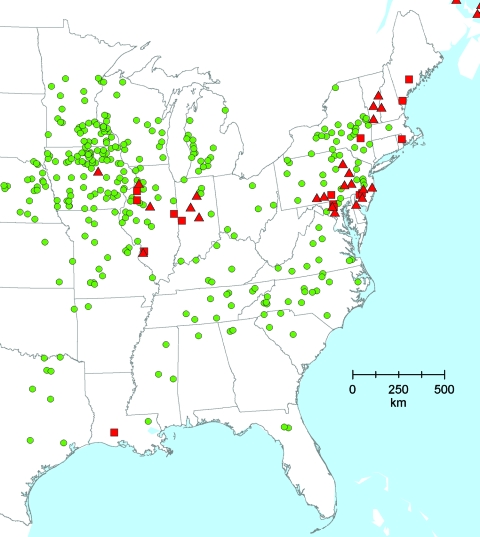
Locations of pastured-pig operations (green dots) and previous records of *Trichinella spiralis* in domestic pigs (red squares) and wildlife (red triangles), United States.

**Figure 2:**
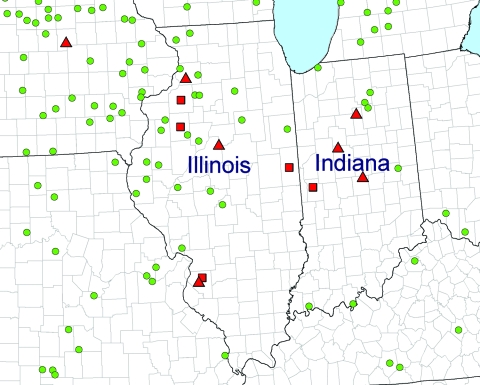
Pastured-pig operations (green dots) and previous records of *Trichinella spiralis* in domestic pigs (red squares) and wildlife (red triangles), Illinois and Indiana.

The databases were converted into map layers within ArcGIS (Environmental Systems Research Institute, Redlands, CA, USA). A basic political boundaries map served as the base map. The western United States is not shown in Figure 1 because the main areas with frequent reports of wild animal and domestic pig *T. spiralis* infections and a prominent pastured-pig industry are the Northeast/Middle Atlantic and the central Midwest. Figure 2 shows an enlargement of the Midwest area to illustrate the ability to more precisely locate risk locations (county level). A GIS analysis, using a program in ArcGIS, was performed to measure the distance between pastured-pig farms and historical occurrences of *T. spiralis* in domestic pigs and wildlife. The program calculates a distance between each pastured-pig farm and the nearest *T. spiralis* point on the map and was run 3 times using the following variables: 1) *T. spiralis* in domestic pigs, 2) *T. spiralis* in wildlife, and 3) *T. spiralis* in both pigs and wildlife (Table).

**Table:** Distances of current pastured-pig operations to locations with past occurrences of *Trichinella spiralis* in domestic pigs or wildlife, United States

Distance to *T. spiralis* infection site and farms, km	Farms near locations of *T. spiralis* in domestic pigs	Farms near locations of *T. spiralis* in wildlife	Farms near locations of *T. spiralis* in both pigs and wildlife	Total
19–50	6	16	6	28
51–100	7	21	20	48

Of the 332 pastured-pig farms mapped, 28 are located within 50 km of documented *T. spiralis* in domestic pigs or wildlife; 6 of these farms are within 50 km of locations with both pig and sylvatic *T. spiralis* infections. An additional 48 pastured-pig operations are within 100 km of *T. spiralis* infection locations.

## Conclusions

Using GIS methods to analyze the risk for *T. spiralis* infection associated with the expansion of pastured-pig production, we identified farms that may be at high risk for the introduction of infection into pigs from reservoir hosts. We base this on the fact that the transmission of *T . spiralis* into sylvatic hosts from infected farms can lead to persistence in reservoir hosts (*2*,*3*) and remain a long-term threat to domestic pigs exposed to such hosts in a pasture/dry lot environment (*2*–*11*). The number of pastured-pig farms and records of *T. spiralis* infections are highest in the Northeast and Midwest. Figure 2 demonstrates the ability through map enlargement to identify associations at the local level. In Illinois and Indiana, at least 10 farms within 50 km of previous *T*. *spiralis* infection in pigs or sylvatic hosts could be identified at the county level. The distances between pastured farms and the locations with recorded foci of *T. spiralis* in wild animals or domestic pigs used in the analysis (Table) are based on the general home ranges for the host species (*14*). For example, raccoons may range up to 3–10 km^2^, red foxes 2–10 km^2^ (with male dispersal up to 80 km^2^), and coyotes up to 50–70 km^2^.

These findings should increase the awareness of pastured-pig producers and state veterinary and public health agencies of this potential problem. Targeted surveillance and management prevention programs need to be established in high-risk areas. The use of GIS tools could also help researchers to conveniently locate transmission foci to investigate the measures needed to prevent infection of outdoor-reared pigs. The database we created on pastured-pig operations is undoubtedly an underestimate of risk because of a lack of a national centralized reporting system for these rearing systems. Furthermore, the infection records are not from a national prevalence survey, which is lacking, but were complied from publications of local surveys and outbreaks (convenience samples). The bias from this method does not, we believe, detract from the objective to introduce the use of GIS tools for identifying foci with potential for *T. spiralis* transmission in outdoor pig-rearing systems. Identification of such foci would provide the opportunity to investigate transmission among wild animals and pigs in agro-ecosystems and the variables that influence transmission, such as climate, pig farm size, herd size, and pig exposure.
